# Costs of adult functional neurological disorders at a tertiary hospital in central South Africa

**DOI:** 10.4102/sajpsychiatry.v29i0.2010

**Published:** 2023-06-20

**Authors:** Leonriche L.C. Christopher, Paul J. Pretorius, Anand Moodley, Gina Joubert, Tracy Arendse

**Affiliations:** 1 Department of Psychiatry, Faculty of Health Sciences, University of the Free State, Bloemfontein, South Africa; 2 Department of Neurology, Faculty of Health Sciences, University of the Free State, Bloemfontein, South Africa; 3 Department of Neurology, School of Clinical Medicine, University of KwaZulu-Natal, Durban, South Africa; 4 Department of Biostatistics, Faculty of Health Sciences, University of the Free State, Bloemfontein, South Africa; 5 Division of Public Health Surveillance and Response National Institute for Communicable Diseases, Johannesburg, South Africa

**Keywords:** functional neurological disorder, neurological disorders, inpatient costs, hospitalisation, FND prevalence, FND demographic variables

## Abstract

**Background:**

Functional neurological disorders (FND) lead to increased care requirements and costs, negatively impacting healthcare budgets. Healthcare expenditure in FND has escalated beyond other neurologic disorders during the past decade.

**Objectives:**

To assess inpatient costs in adults admitted to the neurology ward at Universitas Academic Hospital (UAH) in central South Africa.

**Methods:**

A retrospective observational study with a comparative component was conducted on patients admitted during 2018 and 2019. All FND cases (*n* = 29) and a systematic sample of other neurological disorders were included in the comparison group (*n* = 29). Data were obtained from the Meditech billing system and clinical records.

**Results:**

FND patients accounted for 5.5% of 530 admissions in the neurology ward during the study period. No significant differences regarding daily median cost, age categories, gender or medical comorbidity were observed between FND and the comparison group. However, the length of stay was significantly shorter for the FND patients (median of four versus eight days), translating to approximately half the total costs of patients admitted for other neurological disorders.

**Conclusion:**

The daily median cost was similar for FND and other neurology-related admissions. The lower overall inpatient costs for FND patients were only related to significantly shorter durations of stay, which may reflect new diagnostic approaches resulting from changes in the Diagnostic and Statistical Manual of Mental Disorders, fifth edition (DSM-5) diagnostic criteria. The prevalence of FND was similar to those reported in previous studies conducted at neurology clinics.

**Contribution:**

The study contributes towards better understanding the prevalence and cost of FND in local neurology inpatient care settings.

## Introduction

The symptoms of functional neurological symptom disorder (FND) are inconsistent with the range of clinical manifestations of other neurological, medical or mental disorders.^1^ Clinical findings must show clear evidence of incompatibility between the symptoms and recognised neurological or medical conditions for diagnostic purposes. In the *Diagnostic and Statistical Manual of Mental Disorders, 5th edition* (DSM-5), an identifiable stressor is no longer a prerequisite for the diagnosis. This change should improve diagnostic accuracy in many cases.^1^ Functional neurological disorders are often correctly diagnosed after considerable delays and are a common source of disability in medicine.^2^

Evidence suggests that primary, emergency and inpatient healthcare use by patients with FNDs is high and increases more than for other neurological disorders, with significant cost implications.^3,4,5,6,7^ Functional neurological disorders are of unknown aetiology and often require investigation-intensive hospital admissions.^3,6^ A key concern has been the fear of misdiagnosis, possibly contributing to unnecessary and costly evaluations and inappropriate treatments.^2^ Furthermore, FND is characterised by high rates of psychiatric comorbidity and other medical conditions.^6^ Long-term studies suggest a chronic recurring course with low remission rates and persisting impairment in most patients (31% – 71%).^8,9,10^

Locally, it is unknown if incurred spending for FNDs mimics international study results that indicate higher and increasing costs compared to other neurological diseases.^3,4,5,6,7^ Given the over-stretched healthcare resources and planned implementation of the national health insurance (NHI) in South Africa, knowledge of the cost of FNDs to the healthcare system is essential. Knowledge of the costs incurred for FND patients is pertinent to clinicians working in a resource-constrained environment. There is currently a lack of published data on the local costs of FNDs, and therefore, their contribution to healthcare spending is unknown.

Because of a historical lack of diagnostic clarity, the epidemiology of FND is complex. Although transient conversion symptoms are common, the precise prevalence of FND is unknown.^1^ Before DSM-5, the lack of a clear definition hampered accurate epidemiological findings.^2^ The incidence rate for mixed FND is estimated at 4–12 per 100 000 population per year.^11,12,13,14,15,16,17^ Stone et al.^18^ identified functional and psychological symptoms in 16% of patients referred to neurology clinics over 15 months. Furthermore, up to 30% of patients in their study had symptoms that could not be explained or fully explained based on a known disease.^18^ Low socioeconomic status and female gender have been associated with FND, with women comprising 60% – 75% of FND cases.^2,8^

Functional neurological disorder was previously known as conversion disorder in the *Diagnostic and Statistical Manual of Mental Disorders, 4th edition* (DSM IV)^19^ and was incorrectly assumed to primarily be a psychiatric disorder and was associated with underlying emotional stressors.^20^ Recent abnormal findings in functional brain imaging studies, and the absence of identifiable stressors in many cases, confirmed the clear biological underpinnings of FND.^20,21^ These findings suggest increased brain activity in regions responsible for motor execution and inhibition, including the supplementary motor area and the right temporoparietal junction (TPJ). The right TPJ is responsible for self-agency, hypothesised to be defective in FND. These abnormalities are postulated to result in an exaggerated response to perceived social threats that may result from an over-sensitivity to emotional stimuli.^20^

The objectives of the study were: (1) to establish the prevalence of FND among inpatients admitted to the neurology ward at Universitas Academic Hospital (UAH) in Bloemfontein, South Africa, for the period 01 January 2018–31 December 2019; (2) to describe the demographic profile of inpatients diagnosed with FND at the neurology ward, during the study period and (3) to compare the cost of neurology inpatients with FND and those with other neurological disorders during this period.

## Research methods and design

### Study design

A retrospective observational study with a comparative component was conducted.

### Study population and sampling

For the first objective, all patients aged 14 years and above admitted to the neurology ward of UAH from 01 January 2018 until 31 December 2019 were included as the denominator. All patients who fulfilled the following criteria were included in the numerator for the first objective and the FND group for the second and third objectives: (1) a diagnosis of FND made during the study period; (2) admission to the neurology ward and (3) ≥ 14 years of age. Only patients 14 years and older are admitted to this ward.

All patients diagnosed with FND were included in the study. For the third objective, a comparative group of similar age was randomly selected from 501 patients admitted to the same ward during the study period. A systematic sample of every 12th patient who did not have FND was selected for the comparative group until the numbers in the two groups matched. Patients’ clinical records that did not contain all the required demographic and clinical variables were excluded from the study.

### Data collection

Secondary data were collected from patients’ notes on Meditech, an electronic database containing clinical information for all patients admitted to UAH. The total number of admissions to the neurology ward from 01 January 2018 to 31 December 2019 was recorded. In addition, clinical files of patients admitted to the neurology ward during the period under investigation were reviewed to identify all patients diagnosed with FND. The ICD 10 codes F44.4 (conversion disorder with motor symptom or deficit) and F44.9 (dissociative [conversion] disorder, unspecified)^22^ were searched on the Meditech database.

The patients’ accounts containing all costs incurred during the admission period were retrieved from the revenue section of UAH. The first author entered all demographic, clinical and financial data during July 2020 into a Microsoft Excel data collection spreadsheet designed for this study. The following variables were transcribed on the data form: age, gender, clinical presentation, length of stay, medical comorbidities, psychiatric comorbidities, blood investigations, cerebrospinal fluid investigations, radiological investigations, neurophysiological investigations, medications administered, allied health services received and H classification.

Regarding cost, the Uniform Patient Fees Schedule (UPFS) proposed by the National Department of Health is used for billing all patients. Their H classification determines the amount billed to the patient according to their annual income. The categories for H classification range from H0 to H4 (H0: pensioners; H1: <R70 000 per annum; H2: R70 000–250 000 per annum; H3: >R250 000 per annum; H4: patients with a medical aid). (At the time of preparing this article, the South African rand [ZAR] to United States dollar [USD] exchange rate was 14.64 rand (R) per dollar. Therefore, patients categorised as H1 earned approximately $0.00–$4781.00 per annum, category H2 patients earned $4781.00–$17 076.00 and category H3 patients made >$17 076.00 per annum; information obtained from Currency Converter live rates.^23^ The UPFS is applied by the revenue section of UAH and contains all costs incurred by the hospital. These costs include the ward and professional fees, laboratory fees, professional imaging fees, pharmacy fees, medication fees and allied health service fees.

### Data analysis

Data analysis was conducted by the Department of Biostatistics at the University of the Free State using SAS Version 9.4 (SAS Institute Inc.; Cary, NC). Medians with interquartile ranges (IQR) were reported for the patient cost of FND. Medians and quartiles were used to summarise other numerical variables with skew distributions. Frequencies and percentages summarised categorical variables. Ninety-five percent confidence intervals (95% CI) were calculated for primary outcomes. Groups were compared using chi-squared and Fisher’s exact tests for categorical variables and Mann–Whitney tests for numerical variables.

### Ethical considerations

Approval to conduct the research was obtained from the Health Sciences Research Ethics Committee (HSREC) of the University of the Free State (ethics clearance number UFS-HSD2020/0342/2909). Permission was also obtained from the Free State Province Department of Health. All data were obtained from patients’ hospital account files; therefore, no informed consent was required.

Data were stored safely and securely on a password-protected computer. The Excel spreadsheet was also password-protected to ensure the safety of the data. Data will be retained for five years after completion of the study according to HSREC regulations and facilitate information for subsequent follow-up studies. After five years, data records will be destroyed by the principal investigator.

## Results

Functional neurological disorder accounted for 5.5% (*n* = 29) (95% CI 3.7%; 7.8%) of 530 inpatient admissions to the neurology ward during the period under investigation. The median age reported for FND was 28 years (IQR 19–36), while the median age of the other neurological disorder (non-FND) patients was 34 years (IQR 25–45). The 95% CI for the median difference in age was −4 to 0 years, *p* = 0.046. However, no significant differences in age categories or gender were observed between the groups (Table 1). The length of stay was significantly shorter for patients diagnosed with FND (median four days vs eight days in the comparative group, 95% CI for the median difference of −6 to −1 days (*p* = 0.008).

**TABLE 1:** Demographic characteristics of patients with functional neurological disorders versus patients with other neurological disorders.

Characteristics	FND (*n* = 29)		non-FND (*n* = 29)		*p*-value
	*n*	%	*n*	%	
**Age (years)**					
14–29	16	55.2	11	40.7	0.204
30–49	12	41.4	13	37.9	-
≥ 50	1	3.4	5	17.2	-
**Gender**					
Male	11	37.9	11	37.9	1.000
Female	18	62.1	18	62.1	-
Length of stay in days: median (IQR)	4	2–7	8	4–11	0.008

IQR, interquartile ranges; FND, functional neurological disorders; non-FND, other neurological disorders.

Medical comorbidities were more prevalent in the comparison group, but the difference failed to reach statistical significance (Table 2). The most common medical comorbidity among patients with FND was human immunodeficiency virus (HIV) (*n* = 4; 13.8%), while epilepsy (*n* = 7; 24.1%) was the most common among non-FND. Major depression was diagnosed in 17.2% (*n* = 5) and 10.3% (*n* = 3) of FND and non-FND patients, respectively (Table 3). Symptom presentation in FND patients ranged from paraplegia (*n* = 12; 41.4%), psychogenic non-epileptic seizures (*n* = 6; 20.7%), hemiplegia (*n* = 4; 13.8%), movement disorder (*n* = 3; 10.3%), visual disturbance (*n* = 3; 10.3%) and quadriplegia (*n* = 1; 3.5%). The most common symptoms in the non-NFD group were ataxia (*n* = 7; 24.1%) and visual disturbances (*n* = 5; 17.2%).

**TABLE 2:** Medical comorbidities in patients with functional neurological disorder versus patients with other neurological disorders.

Medical comorbidities	FND (*n* = 29)		non-FND (*n* = 29)		*p*-value
	*n*	%	*n*	%	
Total number of patients with comorbidities*	14	48.3	21	72.4	0.060
Rheumatic fever	1	3.5	0	0	1.000
Obesity	1	3.5	1	3.5	1.000
Diabetes	1	3.5	1	3.5	1.000
Vitamin B12 deficiency	1	3.5	1	3.5	1.000
HIV	4	13.8	6	20.7	0.730
Asthma	3	10.3	0	0	0.237
Epilepsy	2	6.9	7	24.1	0.144
Lung tumour	1	3.5	0	0	1.000
Hypertension	2	6.9	4	13.8	0.670
Hypothyroidism	1	3.5	3	10.3	0.306
Hypercholesterolaemia	0	0	3	10.3	0.237
COPD	0	0	1	3.5	1.000
Osteoporosis	0	0	3	10.3	0.237
Brain tumour	0	0	1	3.5	1.000
Cerebellar disease	0	0	1	3.5	1.000
Idiopathic intracranial hypertension	0	0	1	3.5	1.000
Congenital platelet dysfunction	0	0	1	3.5	1.000
Myasthenia gravis	0	0	1	3.5	1.000
Traumatic brain injury	0	0	1	3.5	1.000
Abdominal tumour	0	0	1	3.5	1.000
Vasculitis	0	0	1	3.5	1.000

FND, functional neurological disorder; non-FND, other neurological disorders; COPD, Chronic obstructive pulmonary disease.

* Some patients had more than one comorbidity.

**TABLE 3:** Psychiatric comorbidities in patients with functional neurological disorder versus patients with other neurological disorders.

Psychiatric comorbidities	FND (*n* = 29)		non-FND (*n* = 29)		*p*-value
	*n*	%	*n*	%	
Major depression	5	17.2	3	10.3	0.706
Intellectual disability	1	3.5	1	3.5	1.000
ADHD	1	3.5	0	0	1.000
Substance use disorder	1	3.5	0	0	1.000
Unspecified psychotic disorder	0	0	2	6.9	0.491
Adjustment disorder	0	0	1	3.5	1.000

FND, functional neurological disorder; non-FND, other neurological disorders; ADHD, Attention deficit/hyperactivity disorder.

Over 86% of participants in each group had laboratory investigations, and over half were referred for imaging studies. No significant differences related to laboratory, radiological or neurophysiological investigations were observed between the groups (Table 4). Involvement of allied health services during admission was similar in both FND and non-FND patients (Table 4).

**TABLE 4:** Diagnostic and treatment modalities in patients with functional neurological disorder versus patients with other neurological disorders.

Diagnostic and treatment modalities	FND (*n* = 29)		non-FND (*n* = 29)		*p*-value
	*n*	%	*n*	%	
Laboratory investigations	25	86.3	28	96.9	0.353
Imaging studies	15	51.7	17	58.6	0.598
Physiotherapy	0	0	2	6.9	0.491
Occupational therapy	5	17.2	3	10.4	0.706
Speech therapy	0	0	0	0	-
Neurophysiological investigations	1	3.5	0	0	1.000

FND, functional neurological disorder; non-FND, other neurological disorders.

The median total expenditure during the admission period was R12781.03 (approximately $873.00) for FND; and R25069.71 (about $1712.00) for the comparative group (*p* = 0.008) (Table 5). Ward admission, medical practitioner fees and pharmacy costs were significantly less for FND. The median cost and IQR were zero for physiotherapy, occupational therapy, speech therapy and neurophysiological investigations. The median total daily cost was R3269.66 (IQR 2654.98–4651.5) in the FND group and R3130.30 (IQR 2506.78–3932.12) in the non-FND group (*p* = 0.604).

**TABLE 5:** Cost in South African Rands of patients with functional neurological disorders compared to other neurological diseases.

Cost categories	FND	Non-FND	*p*-value
**Laboratory investigations**	-	-	0.280
Median	1599.25	2455.78	-
IQR	1338.08–2412.95	1306.43–2986.64	-
**Imaging**	-	-	0.240
Median	222.00	7145.00	-
IQR	0–7516.00	0–8664.00	-
**Ward admission and medical practitioner fees**	-	-	0.004
Median	8012.00	17218.00	-
IQR	4736.00–15582.00	9277.00–24486.00	-
**Pharmacy**	-	-	0.003
Median	76.75	283.24	-
IQR	0–326.60	127.02–875.77	-
**Total**	-	-	0.005
Median	12781.03	25069.71	-
IQR	9302.99–21626.78	13991.04–39391.01	-

IQR, interquartile range; FND, functional neurological disorders; non-FND, other neurological disorders.

Most patients were classified as H1 (low-income category) in both groups, namely 72.4% of FND patients and 86.2% of non-FND patients (Table 6).

**TABLE 6:** Patients’ H classification and amount contributed by the patient according to H classification.

H classification and patient contributed	Group							
	FND (*n* = 29)				Non-FND (*n* = 29)			
	*n*	%	Amount	s.d.	*n*	%	Amount	s.d.
Patient classification according to income (income per annum)								
H1: (R0–70 000)	25	86.2	-	-	21	72.4	-	-
H2: (R70 000–250 000)	0	-	-	-	1	3.5	-	-
H3: (>R250 000)	1	3.4	-	-	1	3.5	-	-
H4: patients with medical aid.	2	7.0	-	-	1	3.5	-	-
H0: pensioners.	1	3.4	-	-	5	17.2	-	-
Prescribed patient contribution in rand per income category								
H1	-	-	217.20	10.10	-	-	266.40	102.30
H2	-	-	0	-	-	-	12752.13	-
H3	-	-	2792.20	-	-	-	9119.08	-
H4	-	-	495.50	700.70	-	-	1947.80	-
H0	-	-	0	-	-	-	0	-

FND, functional neurological disorder; non-FND, other neurological disorders; SD, standard deviation.

## Discussion

The prevalence of FND is complex and has been hampered, until recently, by the lack of a clear definition. The study’s FND prevalence of 5.5% is consistent with the 5% – 6% reported in the literature for referrals to neurology clinics.^1,9^ Universitas Academic Hospital is a tertiary hospital for the Free State Province, with many patients referred from rural areas where investigation-intensive hospital admissions are not feasible. Therefore, patients often need to be admitted for investigations.

Delayed diagnosis, investigation-intensive hospital admissions and re-admissions are cost drivers in FND and increase the risk of iatrogenic harm.^2,3,4,5,24^ Although daily expenditure was similar for both groups, FND patients had a significantly shorter length of stay, contributing to cost savings. The shorter length of stay may reflect the clarification of diagnostic criteria in the DSM-5 and clinicians’ ability to identify neurologic signs specific to FND accurately.

Previous studies^4,25^ have reported a wide age range of onset of FND, while other studies^26,27^ reported a younger onset of FND symptoms. The study’s median age of FND patients was younger than the comparison group. Although unclear at present, this may result from improved diagnostic clarity and earlier recognition by referring primary healthcare clinicians. Paraplegia, the most common clinical feature in our FND patients, represents a particularly robust clinical presentation of FND, which may have contributed to earlier recognition than in other populations. However, paraplegia as the most common presenting symptom is consistent with previous reports.^28^ The relatively young age, lower expenditure and shorter length of stay of FNDs in our study suggest appropriate early recognition and limitation of iatrogenic harm in the local setting. The significantly lower pharmacy cost of FND patients further supports this notion.

As in some previous studies, more women were represented in the FND group.^4,8,26^ Understanding of the higher prevalence in women remains obscure. Some authors have postulated that FND may be related to subconscious defence mechanisms employed more frequently by women in male dominated societies.^8,20,25^ Notably, the gender distribution was similar in the comparison group, and conclusions could not be drawn regarding the predominance of women in the FND group.

This study is the first to examine the prevalence of FND in Neurology inpatients in the Free State. The prevalence of FND is currently unknown, and the study contributes towards a better understanding of the prevalence in tertiary health care settings. The period for which data were collected is comparable to previous epidemiological studies and could be regarded as sufficient.^18^

Most patients in our study were from low-income categories (hospital classification H1), previously associated with FND and the risk of poor health outcomes.^8,26,29^ Patients attending the Universitas Hospital mostly cannot afford private care; therefore, this finding is not surprising. Unfortunately, obtaining data on the prevalence of FND in private healthcare facilities was beyond the scope of this study.

Information on outpatient costs before and after hospitalisation was not collected, and the results of this study should be interpreted with caution. Outpatient services required to manage FND in a multi-professional context could be considerable. Although patients are often admitted for confirmation of the diagnosis, the treatment of FND patients mainly occurs in outpatient settings. Prospective studies should investigate both in- and outpatient costs incurred in managing FND patients.

Including patients in private healthcare facilities would have enhanced demographic variability but make cost comparisons difficult. The results indicate the prevalence of FND among lower-income categories only. Although data on neurophysiologic investigations, allied health and consultation-liaison psychiatric services were often incomplete, it was for both the FND and comparative group and is unlikely to have influenced the cost findings. Because of the retrospective nature of the data collection, the investigator relied on previously documented clinicians’ diagnoses in the neurology wards. Therefore, the investigator could not use standardised diagnostic criteria (e.g. DSM IV, DSM-5, ICD10) to confirm the diagnosis of FND. Literature suggests that FND, when diagnosed by neurologists, are accurate and remarkably consistent, and incorrect diagnoses of FND are unlikely to have influenced study results.^2^

## Conclusion

*Diagnostic and Statistical Manual of Mental Disorders, 5th edition* changes have improved diagnostic clarity and understanding of the prevalence of FND. Functional neurological disorders are common and can be accurately diagnosed by neurologists on the clinical presentation without requiring extended hospital admissions or extensive and costly investigations. Educating students and clinicians about clinical syndromes outside the simplistic mind–body dichotomy should go a long way towards improved empathy, diagnosis, management and limiting iatrogenic harm.^17,30,31,32^
